# Supplementary material for: Axon-specific microtubule regulation drives asymmetric regeneration of sensory neuron axons
Source: eLife. 2025 Feb 24;13:RP104069. doi: 10.7554/eLife.104069 (PMC11850000; doi:10.7554/eLife.104069)
Supplement: Figure 4—source data 1. [file elife-104069-fig4-data1.zip › Figure 4_SourceData3.pdf]

Naive

Spastin

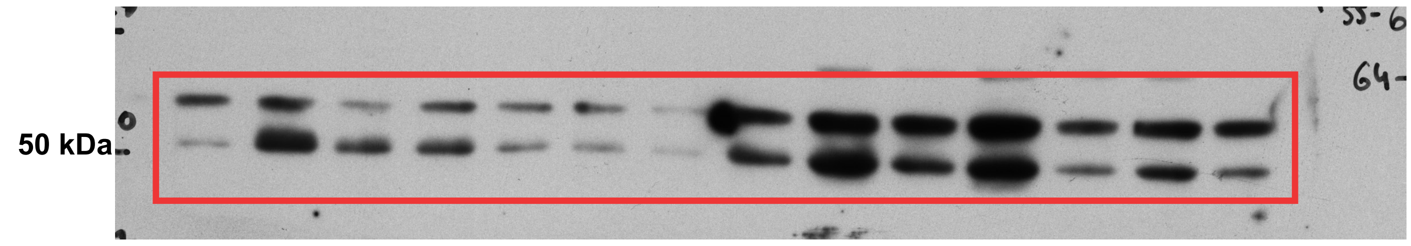

Vinculin

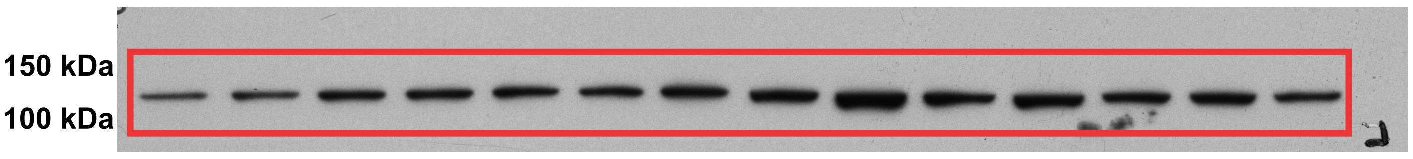

Peripheral nerve Dorsal root

Katanin

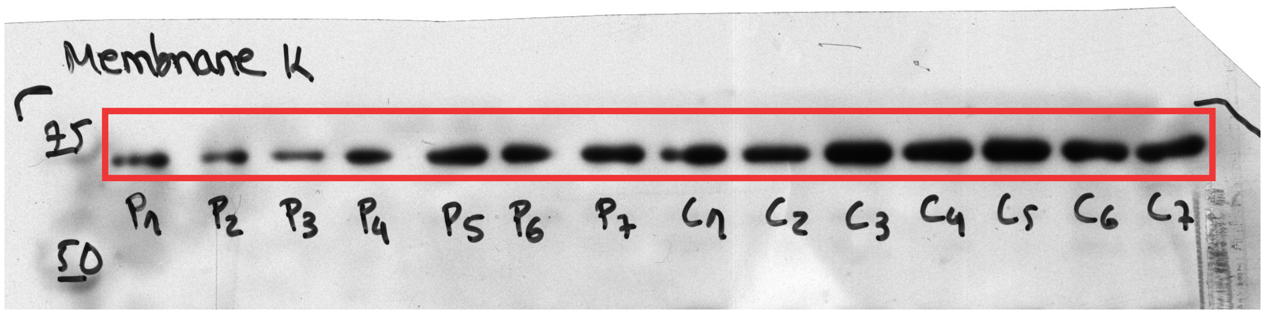

Vinculin

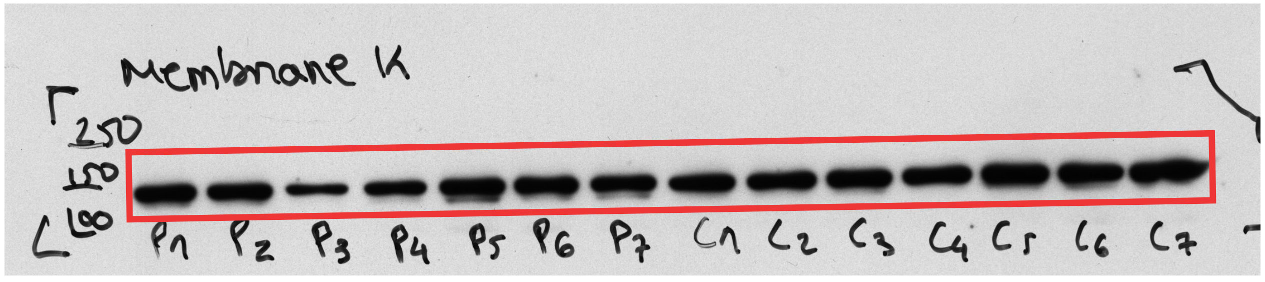

Peripheral nerve Dorsal root

CRMP5

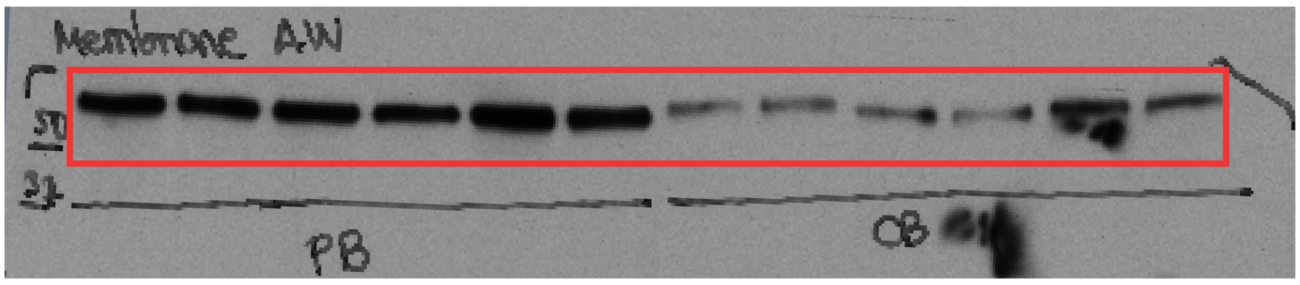

Vinculin

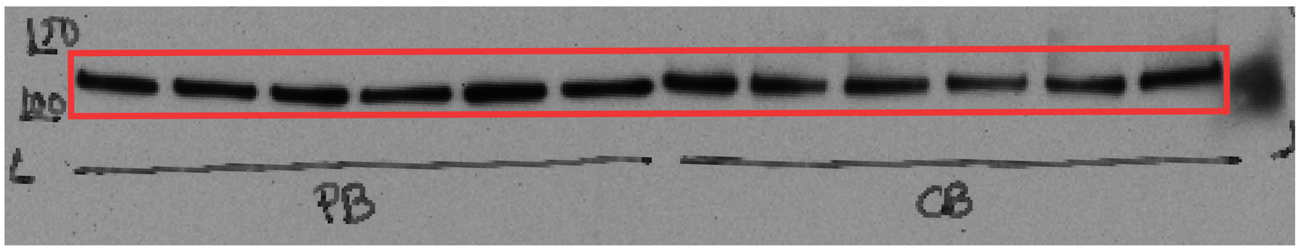

Peripheral nerve Dorsal root

Tau

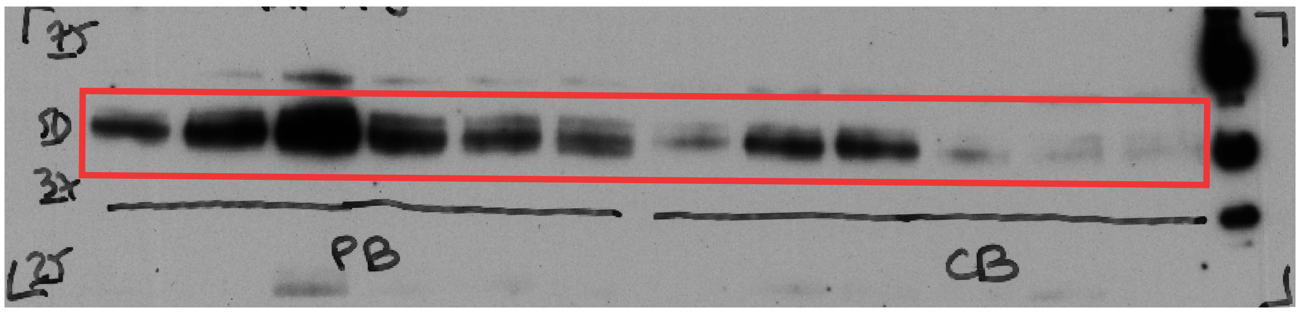

Vinculin

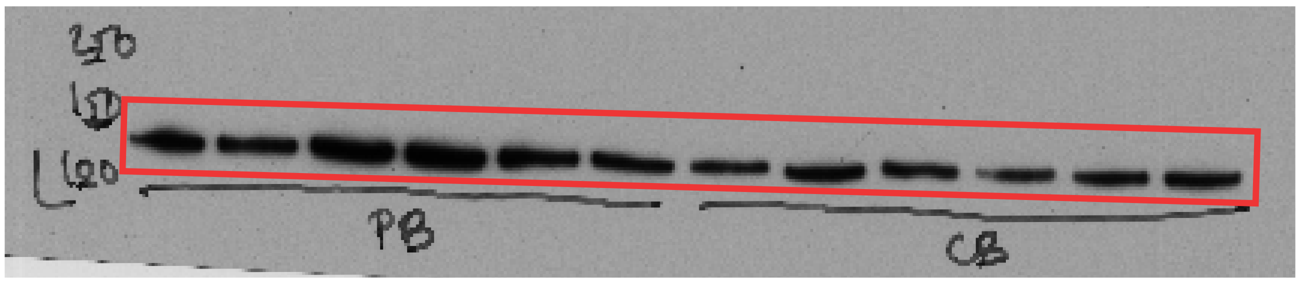

Peripheral nerve Dorsal root

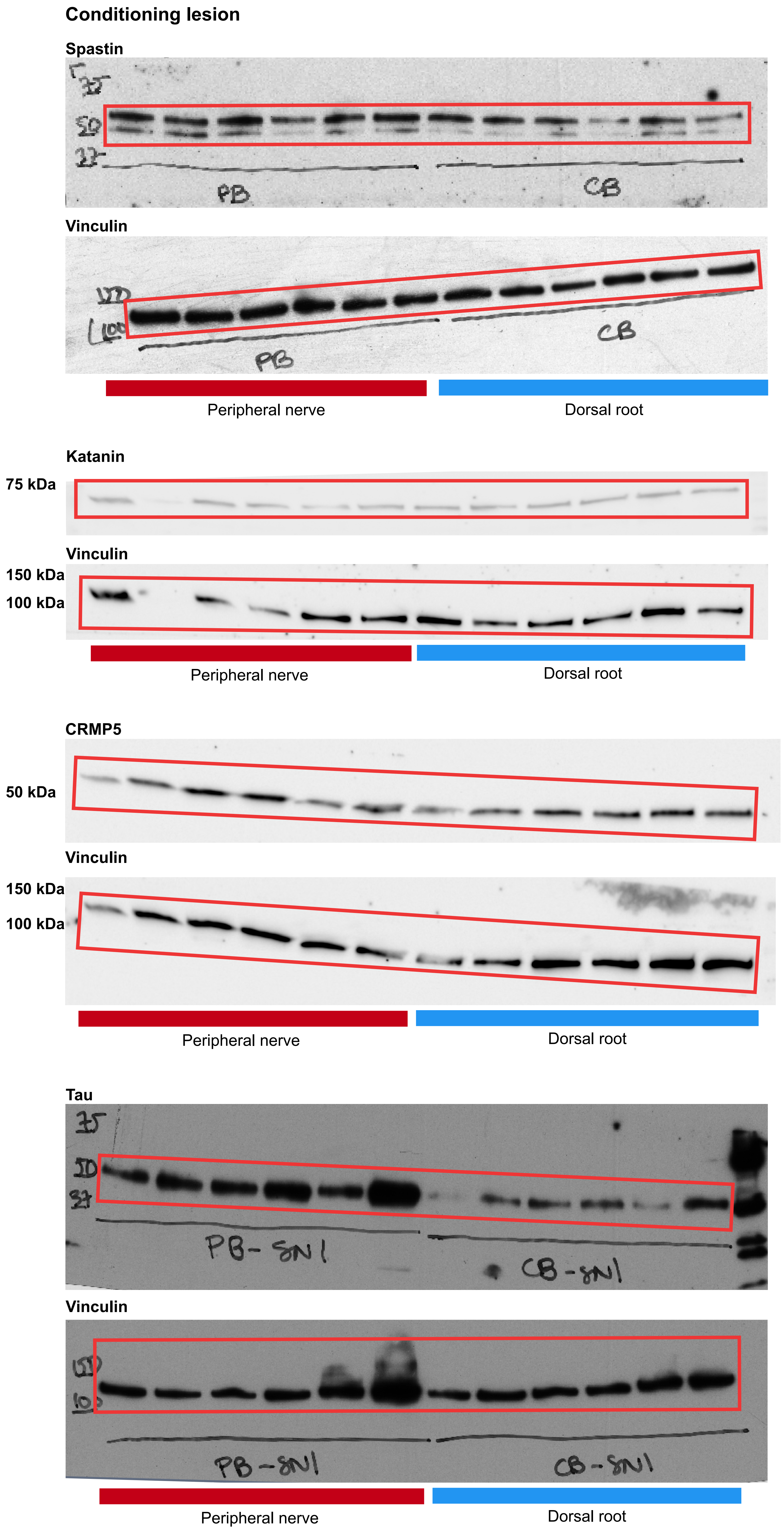

Figure 4, source data 3. Original membranes corresponding to Figure 4, panel B-E (naive) and J-M (conditioning lesion). Microtubule-associated proteins (MAPs), including spastin, katanin, tau and CRMP5 levels, in DRG peripheral nerve and dorsal root. Vinculin was used as a housekeeping protein.Molecular weights writen on the left hand side.
