# Supplementary figures and images for: Axon-specific microtubule regulation drives asymmetric regeneration of sensory neuron axons
Source: eLife. 2025 Feb 24;13:RP104069. doi: 10.7554/eLife.104069 (PMC11850000; doi:10.7554/eLife.104069)

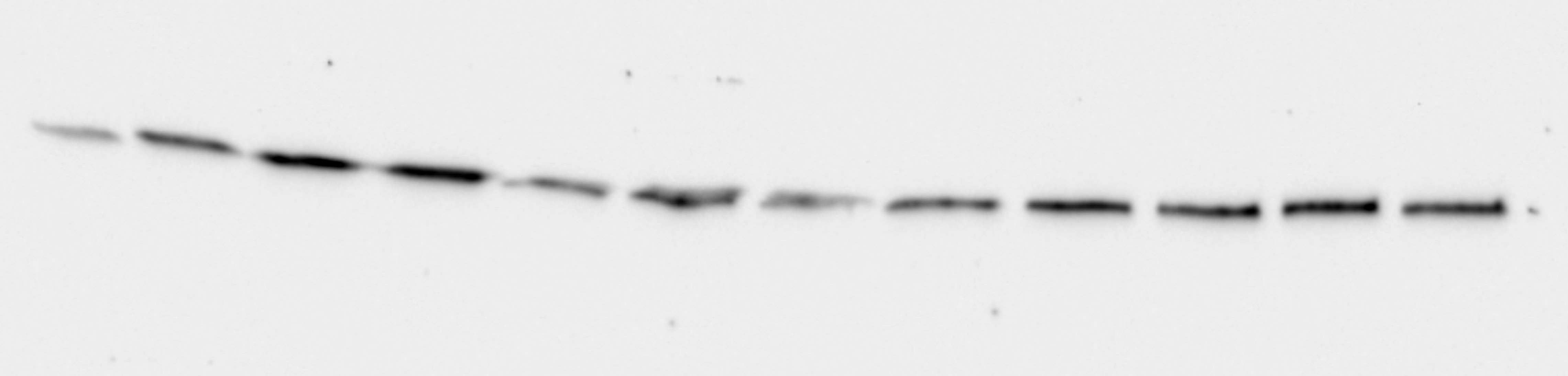

Supplement: Figure 4—source data 2. [file elife-104069-fig4-data2.zip › Figure4_SourceData3/Conditioning_CRMP5.tif]

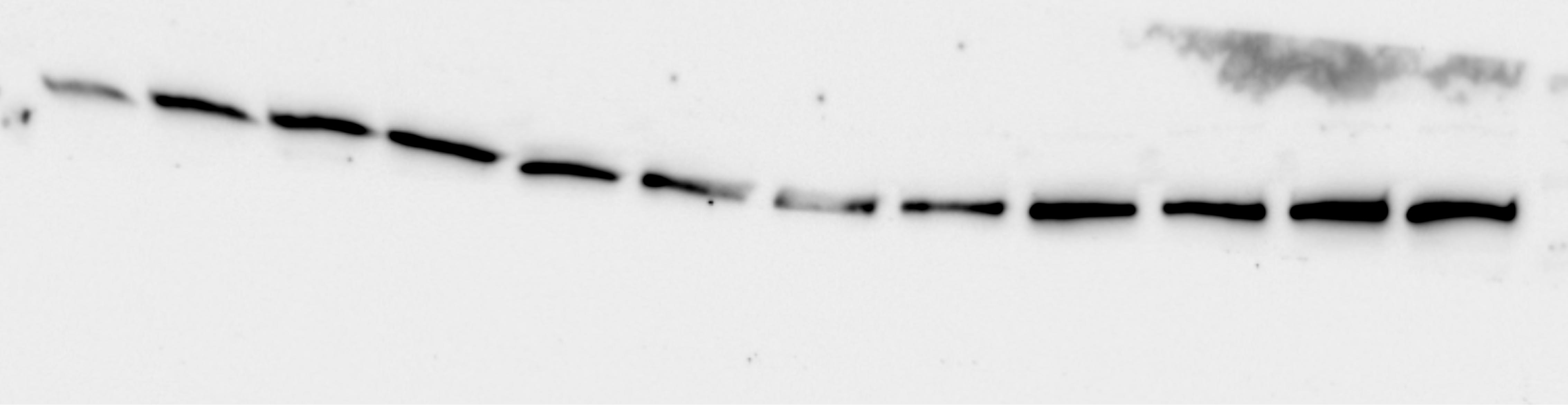

Supplement: Figure 4—source data 2. [file elife-104069-fig4-data2.zip › Figure4_SourceData3/Conditioning_CRMP5_vinculin.tif]

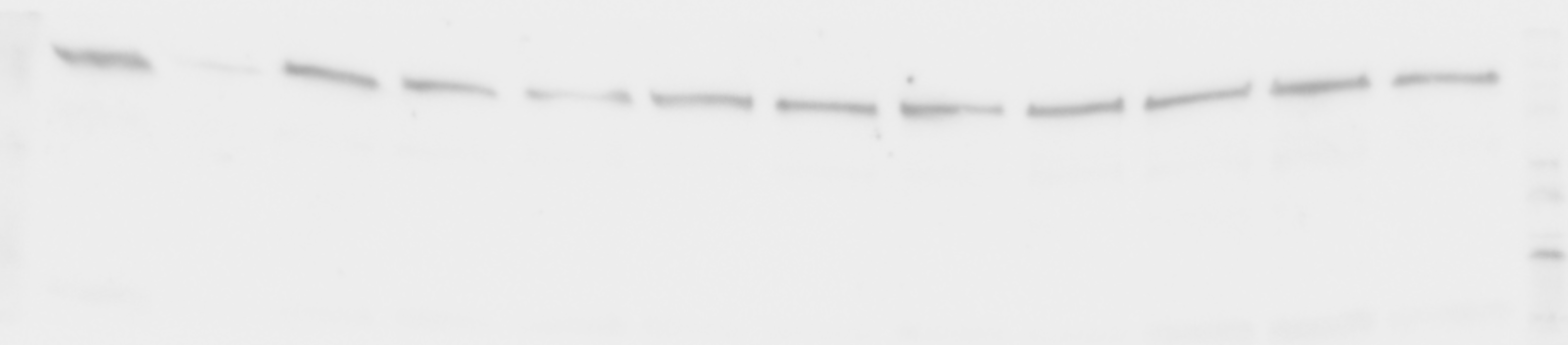

Supplement: Figure 4—source data 2. [file elife-104069-fig4-data2.zip › Figure4_SourceData3/Conditioning_Katanin.tif]

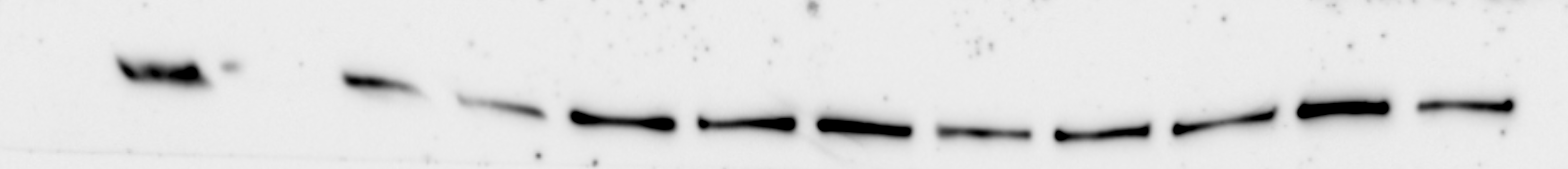

Supplement: Figure 4—source data 2. [file elife-104069-fig4-data2.zip › Figure4_SourceData3/Conditioning_Katanin_vinculin.tif]

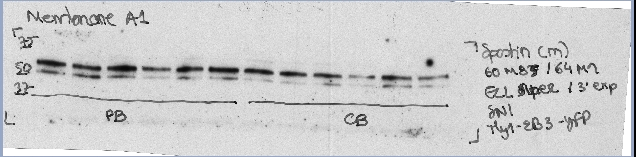

Supplement: Figure 4—source data 2. [file elife-104069-fig4-data2.zip › Figure4_SourceData3/Conditioning_Spastin.tif]

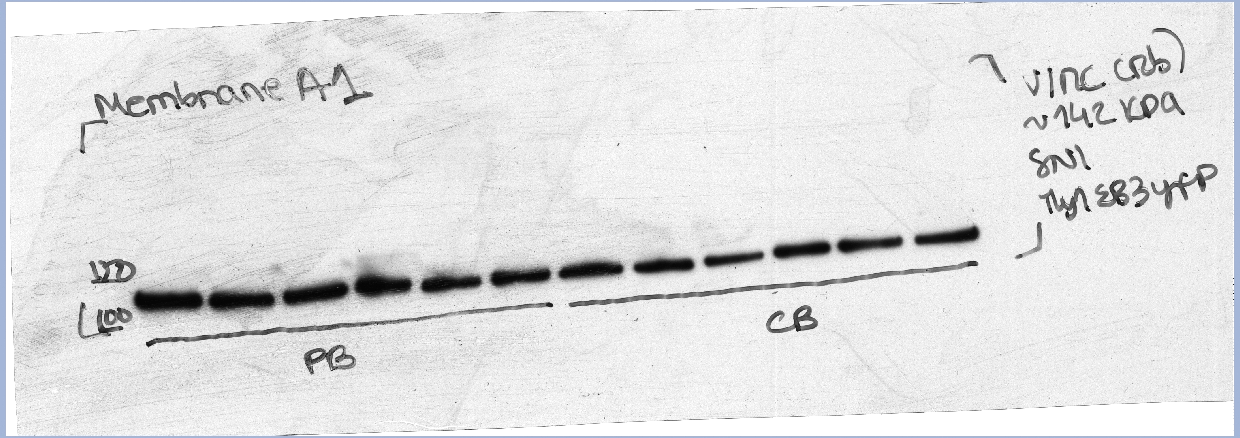

Supplement: Figure 4—source data 2. [file elife-104069-fig4-data2.zip › Figure4_SourceData3/Conditioning_Spastin_vinculin.tif]

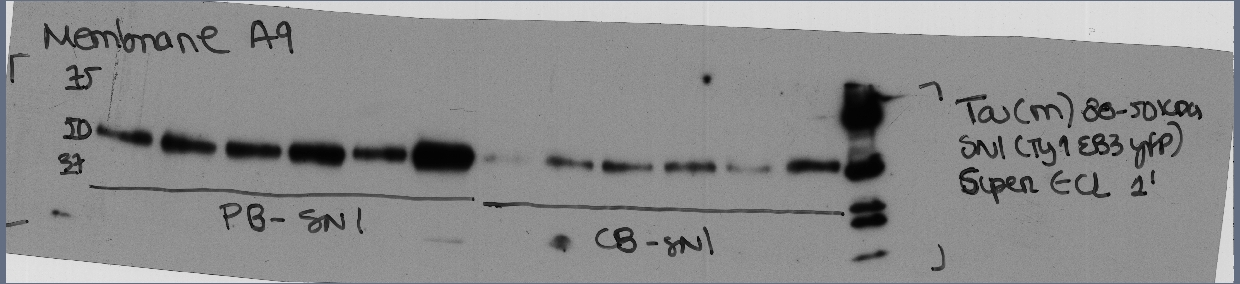

Supplement: Figure 4—source data 2. [file elife-104069-fig4-data2.zip › Figure4_SourceData3/Conditioning_Tau.tif]

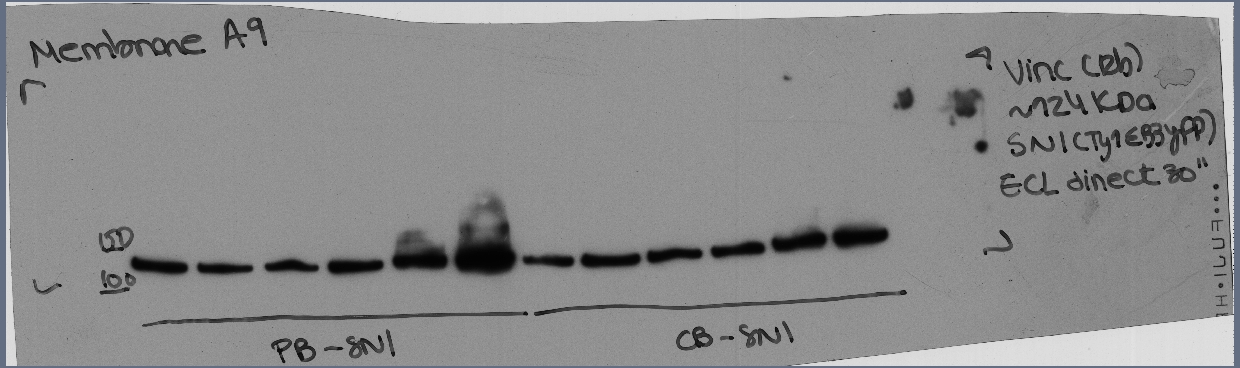

Supplement: Figure 4—source data 2. [file elife-104069-fig4-data2.zip › Figure4_SourceData3/Conditioning_Tau_vinculin.tif]

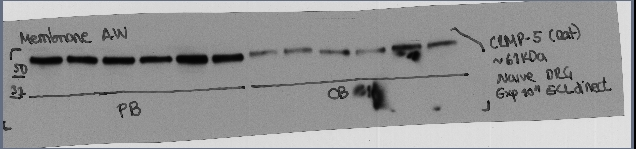

Supplement: Figure 4—source data 2. [file elife-104069-fig4-data2.zip › Figure4_SourceData3/Naive_CRMP5.tif]

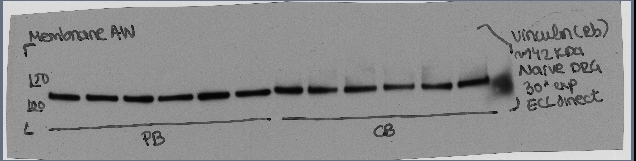

Supplement: Figure 4—source data 2. [file elife-104069-fig4-data2.zip › Figure4_SourceData3/Naive_CRMP5_vinculin.tif]

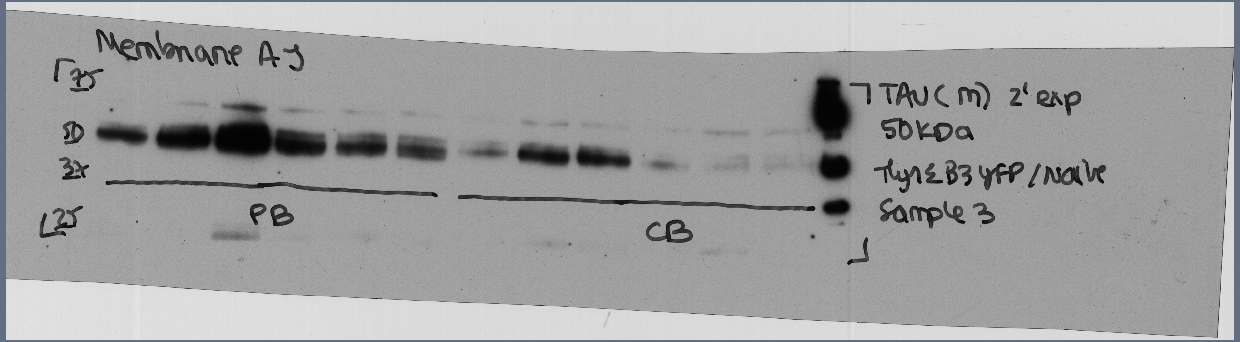

Supplement: Figure 4—source data 2. [file elife-104069-fig4-data2.zip › Figure4_SourceData3/Naive_Tau.tif]

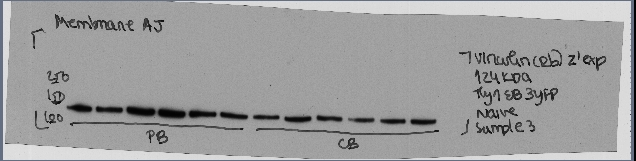

Supplement: Figure 4—source data 2. [file elife-104069-fig4-data2.zip › Figure4_SourceData3/Naive_Tau_vinculin.tif]
