# Supplementary material for: Axon-specific microtubule regulation drives asymmetric regeneration of sensory neuron axons
Source: eLife. 2025 Feb 24;13:RP104069. doi: 10.7554/eLife.104069 (PMC11850000; doi:10.7554/eLife.104069)
Supplement: Figure 4—figure supplement 1—source data 1. [file elife-104069-fig4-figsupp1-data1.zip › Figure 4_FigSupp1_SourceData1.pdf]

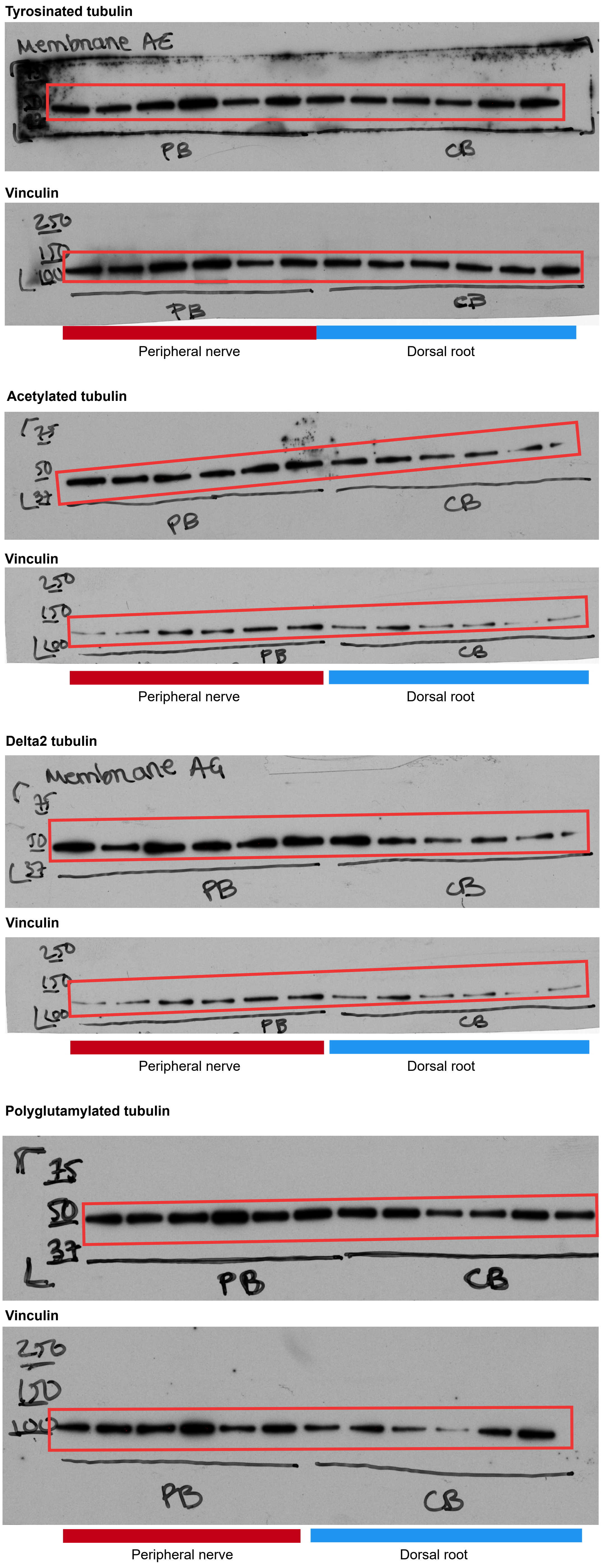

Figure 4, figure supplement 1, source data 1. Original membranes corresponding to Figure 4, figure supplement 1. Tubulin post-translational modifications, including tyrosinated, acetylated, delta2, and polyglutamylated tubulin levels, in DRG peripheral nerve and dorsal root. Vinculin was used as a housekeeping protein. Molecular weights writen on the left hand side.
